# A numerical solution to the effects of surface roughness on water–coal contact angle

**DOI:** 10.1038/s41598-020-80729-9

**Published:** 2021-01-11

**Authors:** Chong Li, Jian Zhang, Jun Han, Banghua Yao

**Affiliations:** 1 School of Mines, Key Laboratory of Deep Coal Resource Mining, Ministry of Education, China University of Mining and Technology, Xuzhou, 221116 Jiangsu People’s Republic of China; 2 School of Safety Science and Engineering, Henan Polytechnic University, Jiaozuo, 454003 People’s Republic of China; 3 Laboratory of Mine Earthquake Monitoring and Prevention, Jiangsu Province, School of Mines, China University of Mining & Technology, Xuzhou, 221116 Jiangsu People’s Republic of China; 4 State Key Laboratory Cultivation Base for Gas Geology and Gas Control, Henan Polytechnic University, Jiaozuo, 454003 People’s Republic of China; 5 State Collaborative Innovation Center of Coal Work Safety and Clean-Efficiency Utilization, Henan Polytechnic University, Jiaozuo, 454003 People’s Republic of China

**Keywords:** Environmental sciences, Natural hazards

## Abstract

Coal dust is a great threat to coal mine workers' health and safety in coal mine production. Wet dust removal is one of the effective dust removal methods. As a solid, coal has different rough surfaces, which have a certain effect on the wetting effect of coal. In this paper, three coal samples with different surface wettability are used as the research objects. Phase-field interface tracking method is used to simulate the wetting of droplets on rough surfaces. From the simulation results, it can be concluded that the influence of the rough interface on the contact angle of the droplets is in accordance with the change rule described in the Wenzel model. As the roughness increases, the contact angle of the hydrophilic lignite surface gradually decreases. As the roughness increases, the contact angle of hydrophobic coking coal gradually increases. The change trend of the contact on the surface of weakly hydrophilic anthracite coal is the same as that of lignite. Due to the local and global differences, the contact angles obtained from the numerical model are slightly different from the values calculated from the Wenzel model.

## Introduction

Coal dust is fine particles that generates in the production process of coal mines. Its hazards are diverse and one major influence is contaminating occupational environment and affecting health of coal mine workers^1^. During 2009–2018, 235,553 cases of pneumoconiosis and other coal dust related respiratory diseases were reported in China, accounting for 87.16% of whole occupational diseases^2^. How to effectively remove coal dust is a problem that coal mine operators need to solve. Wetting coal is one of the most widely used methods for dust mitigation, including water spraying for airborne dust and water infusion in coal seam^3^. Wetting process refers to the process of replacing air on a liquid or solid surface with a water solution. At present, the influence of coal surface physical properties on coal wetting effect is mostly to study the particle size of coal dust. Chemical composition of coal was found to have influences on coal wettability and the contact angle becomes larger as the coal rank increases^4^. The fractal theory to was used to analyze the influence of coal surface structure on coal dust wettability^5^. The results showed that the wettability of coal dust became worse with the increase of particle size fractal dimension. The most obvious case is that when the fractal dimension is greater than 2.5, the contact angle decreases as the dimension increases, and eventually stabilizes at a certain value. When analyzing the coal dust surface wetting mechanism, the smaller coal dust was found to have larger pore volume and more stable cohesion of coal dust with air and coal surface is more difficult to be wetted^6^.

In terms of numerical simulation applied in wetting process on a solid matter surface, the molecular dynamics simulation method was used to study the wetting behavior on the nano-level rough model, and found that under the same roughness value, the shape of the rough structure has little effect on the contact angle, and the wetting models were in accordance with the Wenzel contact model^7^. However, as the roughness of the hydrophobic surface increases, the contact angle will stabilize at a value. The molecular dynamics was used to simulate the wetting of aluminum droplets on a rough surface composed of amorphous carbon and graphite^8^. The simulation shows that the wetting state will change from Cassie Type changed to Wenzel type, as the spacing increases. The lattice Boltzmann simulation method was used to study the influence of the microstructure of the hydrophobic surface on the wetting^9^. The simulation results showed that the hydrophobic surface can be wetted as the height increases. The study also compared the influence of gas–liquid ratio on the contact angle, and found that the gas–liquid ratio has the largest contact angle within a certain range. The effect of surface micro/nano structure on wetting process was studied through the simulation of lattice Boltzmann method^10^. It is found that when the droplet size exceeds the size of the microstructure, the contact angle can be calculated by the classic Cassie equation. When the sizes of the two are almost the same, the contact angle cannot be simply calculated by the Cassie equation.

Under different mining conditions or stress states, coal presents a surface with different roughness, for example, the surface of drilling and excavation, due to the different coal quality and operation mode will show different morphology. It will affect the contact area of water on the coal surface, and thus affect the wetting effect of water on coal. Besides chemical composition of coal surface, coal surface physical property like roughness or morphology can also play an important role in wetting process. However, there is a slight lack of research on the influence of coal surface roughness on wetting, and there are fewer studies on numerical simulation. As a conventional research method, numerical simulation has particularly mature simulation methods and simulation software. When studying the roughness of the coal surface, numerical simulation can ensure the uniformity of the roughness value, and the influence of roughness on the wetting of the coal surface can be studied qualitatively and quantitatively. Therefore, the purpose of this paper is to study the effect of roughness on the wetting of the coal surface via a numerical solution.

## Coal sample selection and natural fracture roughness analysis

### Coal sample selection

Three presentative coal samples were selected, lignite from Hami, coking coal from Anyang, and anthracite from Zhaogu, which have hydrophilic, weakly hydrophilic and hydrophobic properties. Some important parameters of the coal sample, such as the proximate analysis and the hardness coefficient *f*, were tested according to the methods specified in the national standards GB/T 212-2008 and GBT 8208-1987. The results are shown in Table 1.

**Table 1:** Proximate analysis and firmness coefficient of the coal samples.

Coal sample	Proximate analysis			Hardness coefficient
	Moisture (*M*_ad_/%)	Ash (*A*_ad_/%)	Volatile (*V*_adf_/%)	
Hami lignite	5.94	10.05	33.61	0.78
Anyang coking coal	0.57	12.23	23.17	1.12
Zhaogu anthracite	2.98	15.55	7.98	1.92

### Roughness of natural fracture surface

**Figure 1:**
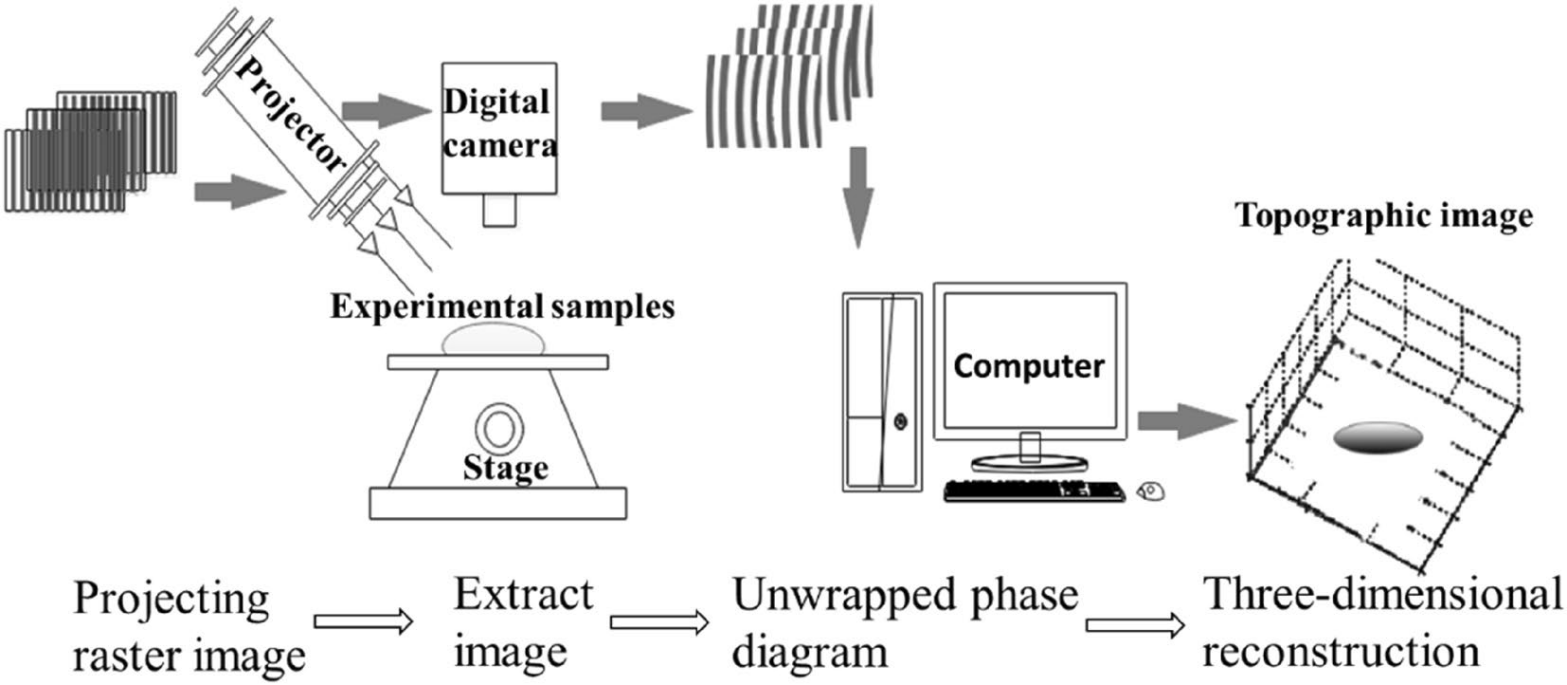
Principle of fringe projection and phase-shifting technology.

The surface roughness is measured using the optical contact Angle 3D morphology coupling instrument produced by Biolin Scientific. The roughness measurement uses the 3D Topography Module, which is composed of a sample platform, a grating projector, a digital camera, and an information processor. The 3D Topography Module is based on Phase Measurement Profilometry (PMP). This technology uses a projector to project a number of sinusoidal grating projections on the surface of the measured object, and the sampling camera collects the grating projection images. Because the collected information is modulated by the measured object, it is necessary to obtain the wrapping information of the height and the position through the demodulation algorithm, and use the unwrapping algorithm to unwrap to obtain the unwrapping information of the position. Finally, through the conversion relationship between the position and the height, the height data of the object surface is obtained^11^. The process of the experiment is shown in Fig. 1.

**Figure 2:**
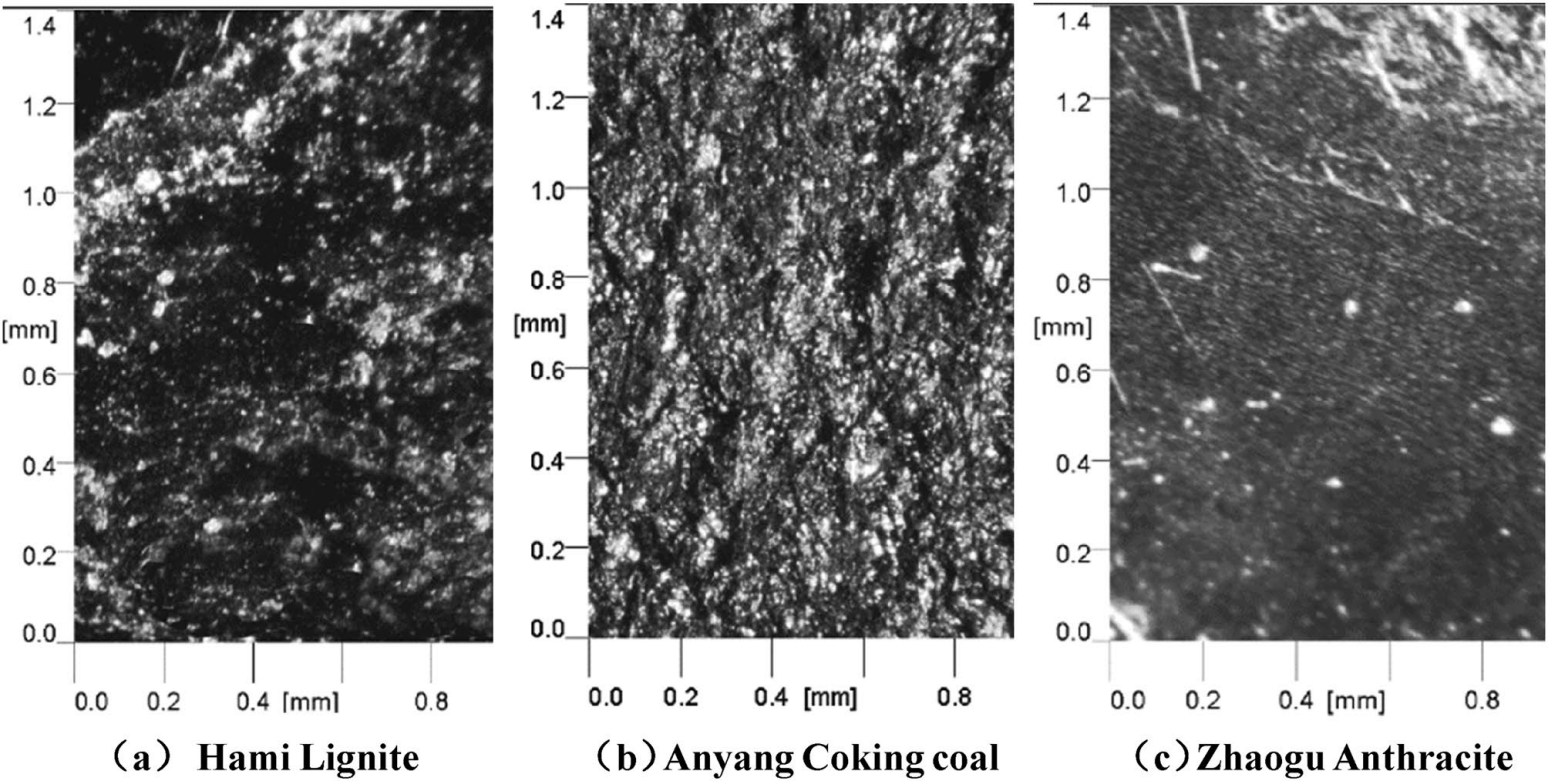
Optical images of the three coal samples.

**Figure 3:**
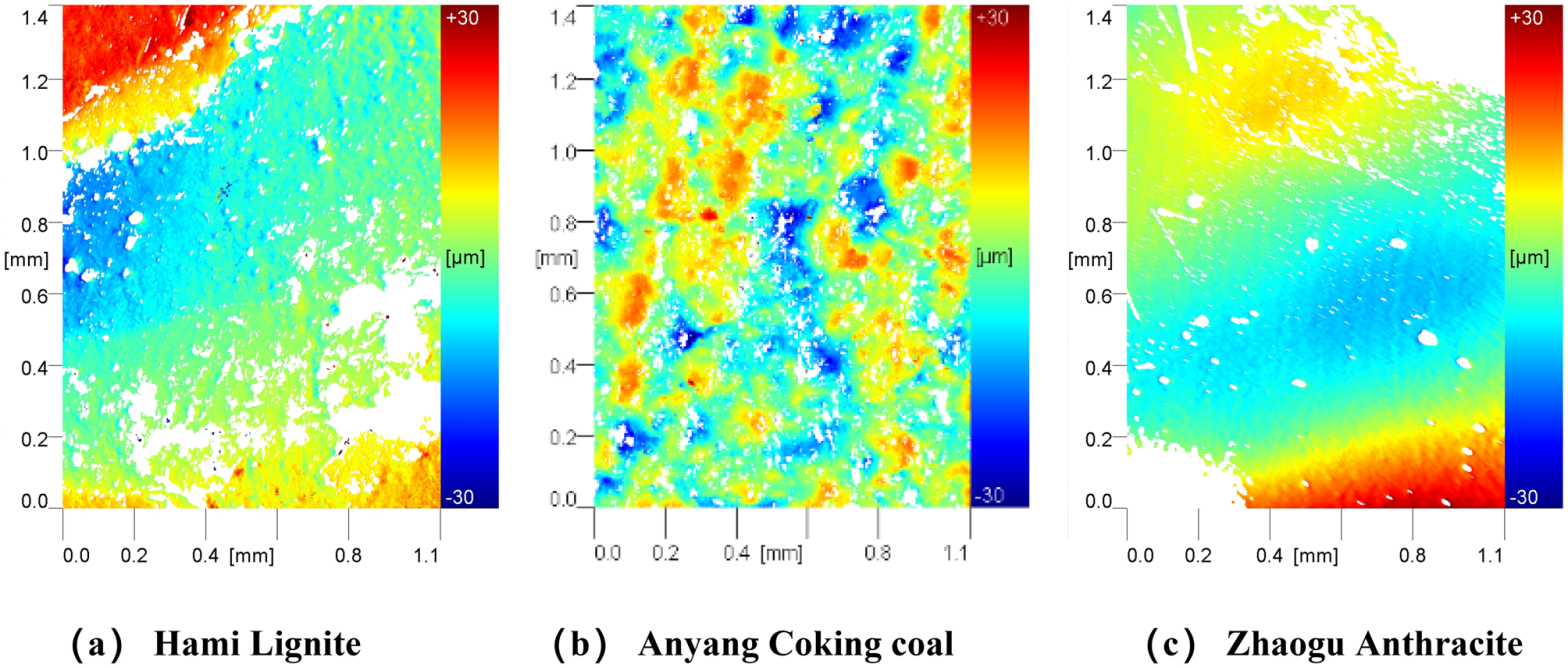
2D reconstructed topography images of the three coal samples.

**Figure 4:**
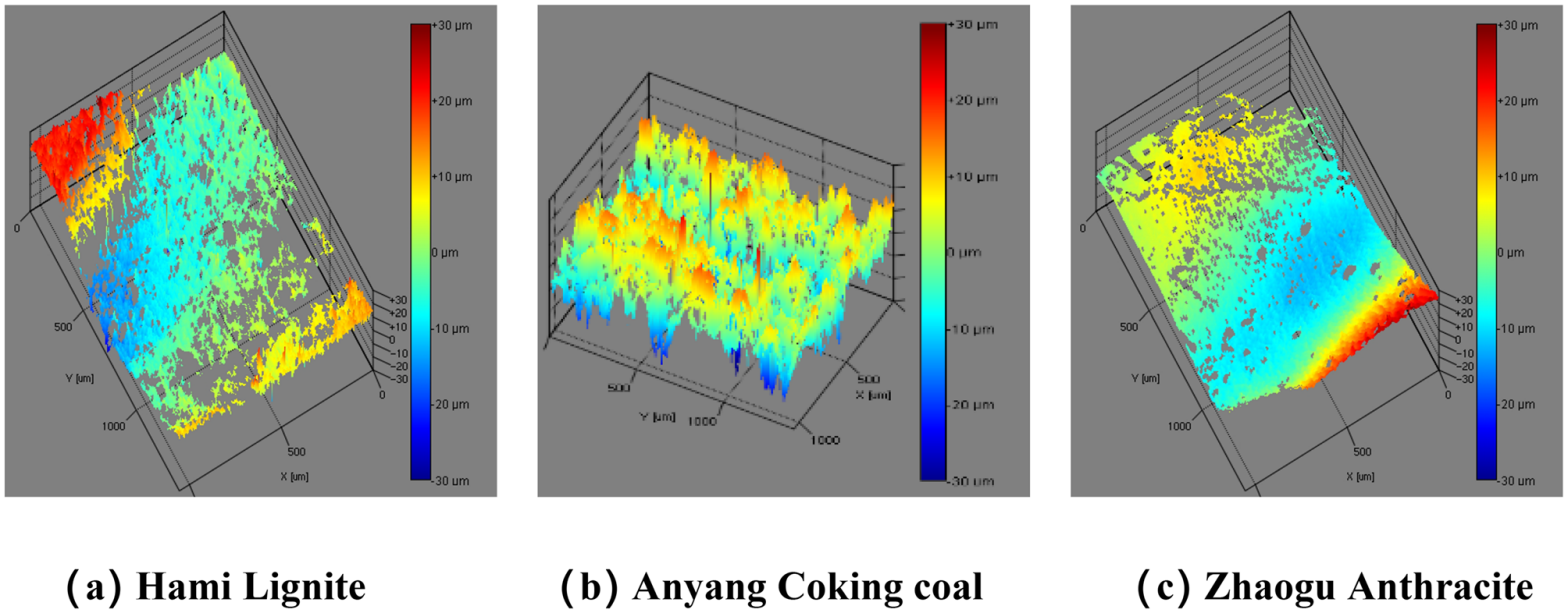
3D reconstructed topography images of the three coal samples.

The morphology and roughness of the natural fracture surfaces of the three coal samples were measured on relatively flat fracture surface to ensure convenient measurement. For each coal sample, three surfaces were selected to be tested within the area range of 1.4 mm × 1.0 mm (Fixed measuring area of equipment), and each surface was scanned five times, and then use the mean square roughness value as the evaluation index to analyze the difference in surface morphology and roughness of different coal samples. Figure 2 shows the optical images of the three coal samples. Figures 3 and 4 presents the reconstructed 2D topography and 3D topography of the fractured surfaces of the three coal samples respectively. Due to the reflection of coal sample, some data is missing, and the missing place will be displayed as blank.

According to the above morphology measurement, the roughness of the natural fractures of the three coal samples are Hami lignite R_q_ = 6.54 μm, Anyang coking coal R_q_ = 5.14 μm, and Zhao anthracite R_q_ = 3.52 μm (where R_q_ is the mean square roughness). From the perspective of coal hardness coefficient, different coal samples have natural fracture surfaces with different roughness.

## Numerical model

### Theoretical model

The concept of contact angle was proposed to quantitatively characterize the wettability of solid surfaces. Generally the surface with a contact angle of less than 90° is defined as a hydrophilic surface, and the surface with a contact angle of greater than 90° is deemed a hydrophobic surface^12^.

The contact angle formulation on the ideal homogeneous and smooth surface was derived by the Young's equation^13^, which relates the intrinsic contact angle to the surface tension between the three phases.

1 $${ \gamma_{sv}}{ - }{\gamma_{sl}} = {\gamma_{lv}}\cos \theta .$$

Contact angle $$\theta $$:

2 $$ \theta = acrcos\frac{{{\gamma_{sv}}{ - }{\gamma_{sl}}}}{{{\gamma_{lv}}}}.$$

Young's equation can be used to predict the ideal contact angle of absolute smoothness, but it is limited to characterizing the contact angle of a droplet on an ideal solid surface. The smaller the contact angle, the better the wettability, and the larger the contact angle, the worse the wettability.

The wettability of the real solid surface was studied and introduced the surface roughness coefficient, namely the ‘roughness factor (*λ*)’^14^. Wenzel also corrected the Young's equation to establish a model of the liquid completely wetted in the local groove area, the equation is written as:

3 $$ cos{\theta_r} = \lambda cos\theta . $$

or

4 $$cos{\theta_r} = \lambda \frac{{{\gamma_{sv}}{ - }{\gamma_{sl}}}}{{{\gamma_{lv}}}}{ = }\lambda cos\theta .$$

where $\theta_r$ is the apparent contact angle, *λ* is the ratio of the actual surface area to the projected area and is always greater than 1. Therefore, in combination with Eq. (3), it is known that the absolute value of the contact angle cosine function formed by the interaction between a real solid surface and a droplet is larger than that of an ideal smooth solid surface. When the solid surface is hydrophilic (contact angle < 90°), the apparent contact angle will decrease as the solid surface roughness increases and the hydrophilicity is better. If the solid surface is hydrophobic (contact angle > 90°), the apparent contact angle will increase with the solid surface roughness. However, it was also found that the hydrophilic surface becomes hydrophobic under certain conditions and it was difficult for Wenzel to explain this phenomenon^15^.

In the study of surface wetting, it was found that during the wetting process of rough solid surfaces, the liquid did not completely enter the surface gaps, and there was still a certain amount of air under the droplets, and the droplets were suspended between the air and the solid composite contact surface^16^. They analyzed from the perspective of thermodynamics and proposed the following equation:

5 $$ cos{\theta_r} = {f_1}cos{\theta_1} + {f_2}cos{\theta_2}$$

where ${\theta_r}$ represents the apparent contact angle of the composite contact surface, ${\theta_1}$ and ${\theta_2}$ represent the intrinsic contact angle of the two media, ${f_1}$ and ${f_2}$ represents the proportion of the two media at the composite interface, ${f_1}{ + }{f_2}{ = }1$.

When the composite contact surface is composed of air and solid, since the contact angle of liquid and air is 180°, Eq. (5) can be simplified to:

6 $$ cos{\theta_r} = {f_1}(1 + cos{\theta_1}) - 1 $$

When the composite contact surface is composed of liquid and solid and air is completely replaced by liquid droplets. Since the contact angle between liquid and liquid is 0°, Eq. (5) can be abbreviated as:

7 $$ cos{\theta_r} = {f_1}(cos{\theta_1}{ - }1) + 1 $$

Equation 7 can be deemed a supplement to Wenzel's equation. The Cassie-Baxter model can explain the contact angle of rough surfaces. However, in actual situations, it is difficult to determine the composite contact surfaces ${f_1}$ and ${f_2}$.

Because wetting is the interaction in the gas–liquid–solid interface, the key to the simulation is to track the three-phase (gas–liquid–solid) interface. There are currently several methods for phase interface tracking, such as Volume of Fluid (VOF)^17^, Lattice Boltzmann Method (LBM)^18^, Particle-in-cell method (PIC)^19^, Level Set method^20^, Phase Field method^21^. The phase field method has practical physical meaning, which is based on the kinetic process of thermodynamics. In order to characterize the changes of moving objects in time and space, $\varphi (r,t)$ variable is introduced, $r$ represents space, and $t$ represents time. $\varphi { = - }1$ indicates the liquid phase zone, $\varphi { = }1$ indicates the solid phase zone, and a value between -1 and 1 indicates the solid–liquid two-phase zone. This method also uses the Cahn–Hilliard equation^22^ to control the phase field change, which is more suitable for the calculation of microfluidic motion and has high calculation accuracy. Therefore, this paper chooses the phase field method to numerically simulate the wetting of water droplets on the coal surfaces.

### Physical model

**Figure 5:**
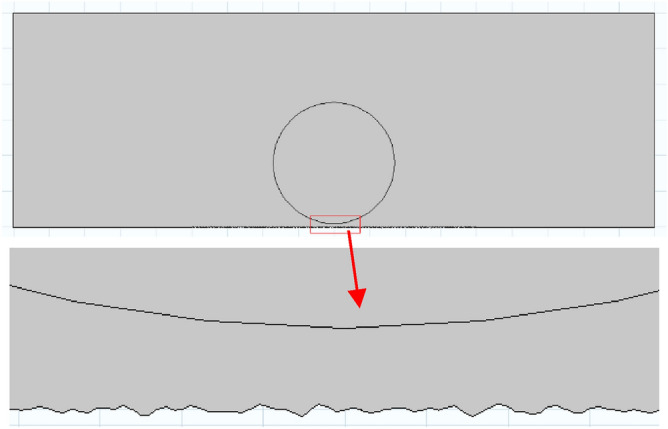
Geometric model of the simulation.

**Figure 6:**
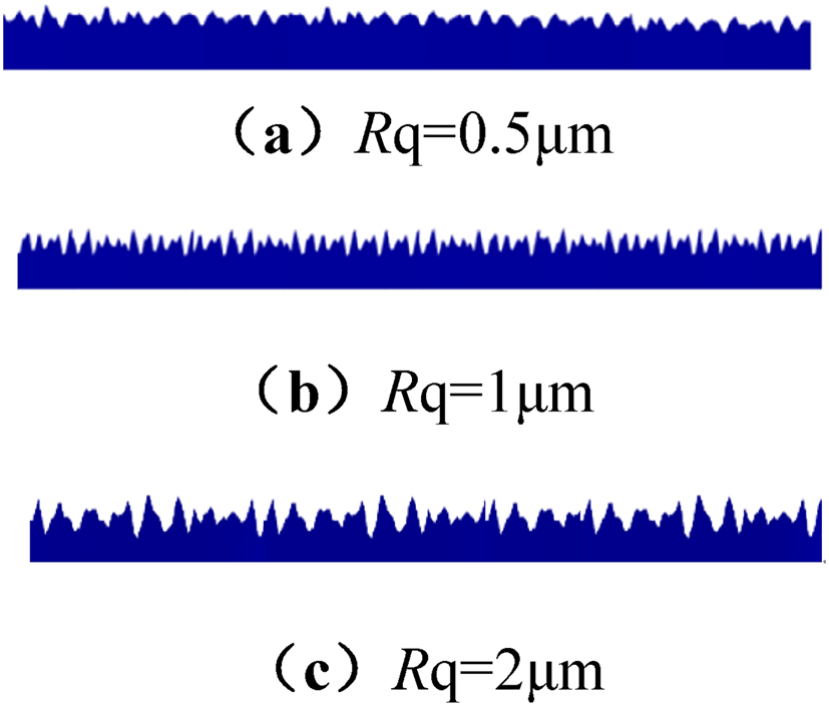
Diagram of different roughness surfaces.

The simulation content is the process of wetting and spreading a droplet on a rough solid surface in an air atmosphere. The established model is a two-dimensional model, the calculation area is 18 mm × 6 mm, the drop is in the center, and the volume is 10 μL (close to the size of the experimental drop). The geometric model is shown in Fig. 5. The roughness surface is obtained by programming, and the random rough surface curve data points are obtained. This method can set different roughness value curve data through parameter adjustment, and then import the data into the COMSOL Multiphysic geometry to obtain the random rough surface, as shown in Fig. 6.

### Governing equations

The governing equations for simulating the wetting of droplets on rough solid surfaces with COMSOL Multiphysic include mass conservation equations, dynamic conservation equations, interface tracking equations, etc.

Mass conservation equation (incompressible fluid):

8 $$ \nabla \cdot u = 0 $$

Momentum conservation equation considering the surface tension of microfluid:

9 $$ \rho \frac{\partial u}{{\partial t}} + \rho (u \cdot \nabla )u = \nabla \cdot \left[ { - pI + \mu \left( {\nabla u + {{(\nabla u)}^T}} \right] + F{\text{s}}} \right.{ + }\rho g $$

where $\rho$ is the density (kg/m^3^), $\mu$ is the dynamic viscosity coefficient (Ns/m^2^),$p$ is the pressure (Pa),$I$ is the identity matrix,$g$ is the Acceleration of gravity (m/s^2^),$Fs$ is the surface tension of the solid–liquid interface (N).

In the phase field, the surface tension in Eq. (9) can be calculated according to the following equation:

10 $$ Fs = G\nabla \phi $$

where $\phi$ is the phase field parameters, $G$ is the chemical potential (J/m^3^).

Cahn–Hilliard equation: tracing the diffusion interface of two immiscible fluids. The diffusion interface is defined as the area where the dimensionless phase field variable $\phi$ ranges from − 1 to 1. In the simulation process, the Cahn–Hilliard equation is divided into two equations:

11 $$ \frac{\partial \phi }{{\partial t}} + {\mathbf{u}} \cdot \nabla \phi = \nabla \cdot \frac{\gamma \lambda }{{\varepsilon^2}}\nabla \psi $$

12 $$ \nabla \psi { = - }\nabla \cdot {\varepsilon^2}\nabla \phi { + }\left( {{\phi^2} - 1} \right)\phi $$

where $\gamma$ is the mobility (m^3^s/kg), *λ* is the mixed energy density(N), $\varepsilon$ is the interface thickness parameter(m), $\psi$ is the phase field auxiliary variable.

The relationship between mixing energy density and interface thickness and surface tension coefficient:

13 $$\sigma = \frac{2\sqrt 2 }{3}\frac{\lambda }{\varepsilon } $$

Generally set the interface thickness parameter to $\varepsilon = {h_c}/2$, which ${h_c}$ is the characteristic grid size of the area passing through the interface. The mobility $\gamma$ determines the time scale of Cahn–Hilliard diffusion, consider specific requirements when selecting, because this parameter needs to be small enough to ensure that the convection term is not excessively suppressed, and it needs to be large enough to keep the interface thickness constant.

In Eq. (10), $\phi$ is the phase field parameters that ranges from − 1 to 1. The volume fraction of each fluid:

14 $$ {V_{1}} = \frac{{1{ - }\phi }}{2},{V_2} = \frac{1 + \phi }{2} $$

In the model fluid 1 is defined as water and fluid 2 is defined as air.

The density (kg/m^3^) and viscosity (Pa/s) of the two-phase interface mixture:

15 $$ \rho = {\rho_w} + \left( {{\rho_a} - {\rho_w}} \right){V_2}$$

16 $$ \mu = {\mu_w} + \left( {{\mu_a} - {\mu_w}} \right){V_2} $$

where *w* represents water*, a* represents air.

Contact angle control equation:

17 $$n \cdot {\varepsilon^2}\nabla \phi = {\varepsilon^2}\cos \left( {\theta_w} \right)|\nabla \phi |$$

where $n$ is the normal vector, $n = \frac{\nabla \phi }{{|\nabla \phi |}}$. This equation can determine the wettability of the interface.

### Intrinsic contact angle measurement

**Figure 7:**
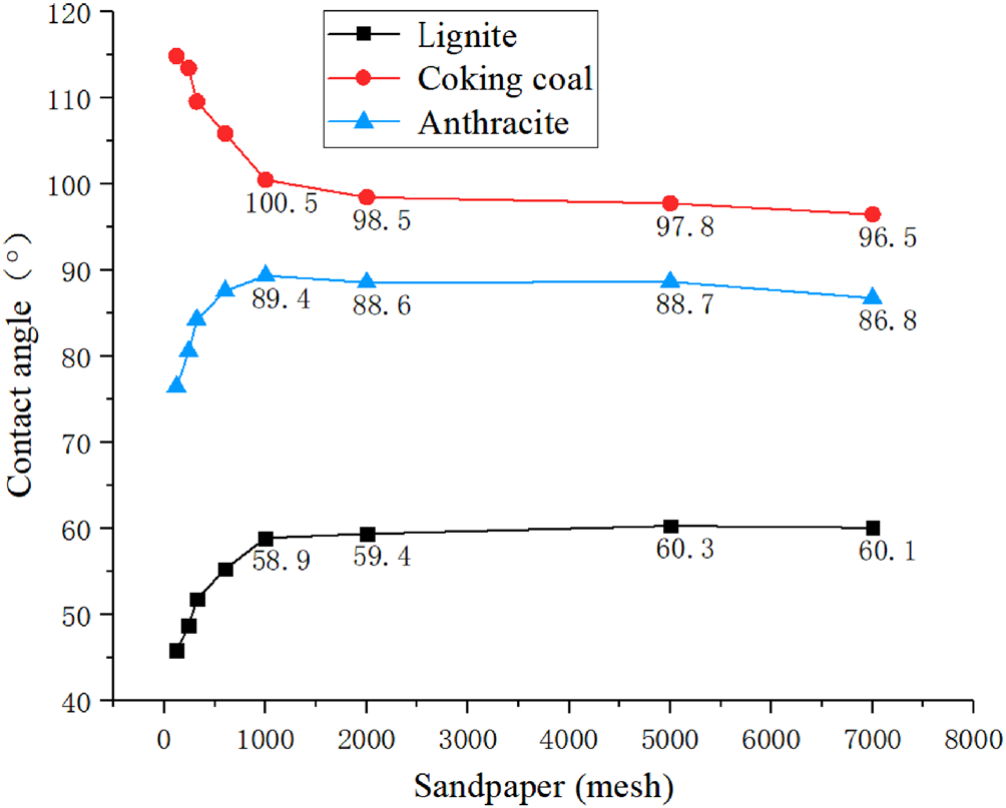
Proximate intrinsic contact angle measurement of the coal samples treated with various sandpapers.

An important parameter of the three coal samples is the intrinsic contact angle. The intrinsic contact angle is the contact angle formed by the substance itself and deionized water under ideal smooth surface conditions. Here we use the sessile drop method to measure the contact angle. A drop is placed on a prepared coal sample surface by using a micro syringe. The profile of sessile drop is photographed for contact angle measurement using a computer program. The intrinsic contact angle can be used to judge the hydrophilicity and hydrophobicity of the substance. Since it is troublesome to add chemical property parameters of solid materials in the simulation, it is simplified to replace the hydrophilic and hydrophobic properties of each coal sample with intrinsic contact. However, it is difficult to achieve an ideal smooth surface, so multiple high-precision sandpapers are used to grind the coal samples to make them close to the ideal smooth. The specific contact angles of the three coal samples are shown in Fig. 7, where the proximate intrinsic contact angle of each coal sample is 60° for Hami lignite, 95° for Anyang coking coal, and 85° for Zhaogu anthracite.

### Simulation conditions

1. Assumptions
  1. There is no reaction between the wall and the liquid, no heat transfer, and the wall temperature is constant.
  2. The fluid is an incompressible Newtonian fluid, and the fluid flow conforms to the laminar flow law.
  3. The liquid has no penetration on the solid surface, only wetting and spreading on the surface.
2. Initial conditions
  The ambient temperature is 298 K (keep at the same temperature as the experiment), the pressure is 101 kPa, the liquid phase is water defined as fluid 1, the gas phase is air defined as fluid 2. The viscosity and density parameters of the fluid derive from the selected material, and the surface tension coefficient is 0.072. The phase interface thickness parameter is determined according to the mesh size, which is one-half of the characteristic mesh size of the phase interface, and the mobility ratio takes the default value.
3. Boundary conditions
  As shown in Fig. 8, the left side of the numerical simulation calculation area is set as the pressure inlet, the right side is set as the pressure outlet, and the pressure value is 0. The upper wall is set as a no sliding wall, and the lower wall is set as wetting wall.

**Figure 8:**
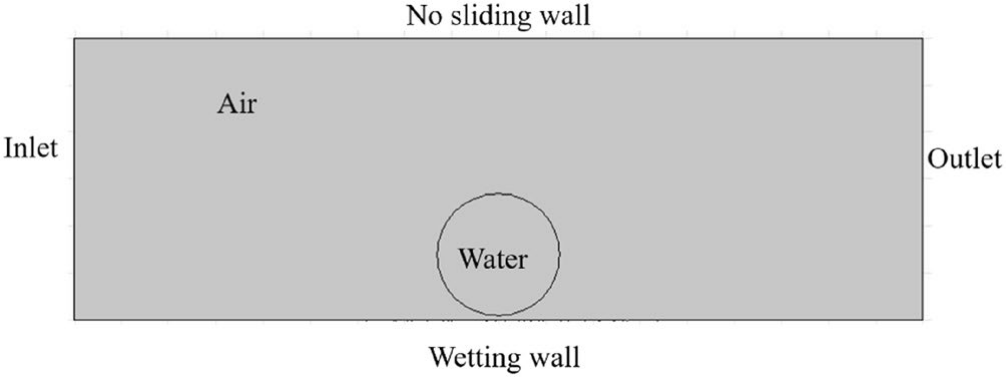
Boundary conditions of the model.
4. Meshing
  The thickness of the mesh division will affect the accuracy and speed of the calculation. In order to improve the calculation accuracy and reduce the calculation time, the droplet boundary and the solid wetted wall are finely divided, and the other areas are divided with coarse mesh, as shown in the Fig. 9. The surface of the droplet and the wetting wall is dense, the smallest unit is 0.0009 mm, the largest unit is 0.06 mm, the maximum unit growth rate is 1.08, the curvature factor is 0.25, and the narrow area resolution is 1. Other calculation areas default to “more fine”, the smallest unit is 0.024 mm, the largest unit is 0.16 mm, the maximum unit growth rate is 1.1, the curvature factor is 1, and its narrow area resolution is 1.

**Figure 9:**
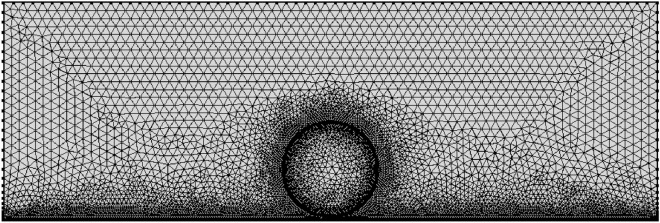
Generated mesh of the model.

## Model validation

**Figure 10:**
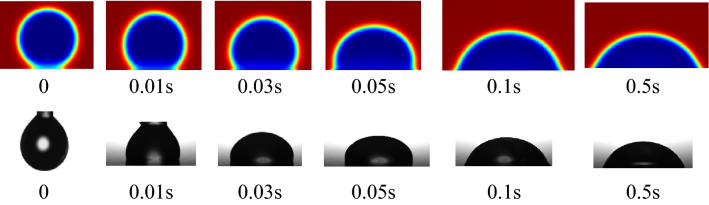
Comparison of simulated and real droplet spreading process.

According to the intrinsic contact angle value and the natural fracture surface roughness value obtained above, the simulation parameters in the COMSOL Multiphysic software are adjusted to simulate the wetting in three different situations. The real contact angle is measured by using the contact angle measurement module in the optical contact angle profiler. The model is validated by calibrating dynamic water droplet spreading process and by comparing contact angles till the steady state. The real and simulated droplet spreading processes on the Hami lignite surface are shown in Fig. 10. The processes are completed in a very short time and they are almost consistent during the time.

Comparison of contact angle between real experiment and simulation in the static state is given in Table 2. Roughness of the model is obtained by substituting the roughness value obtained from the topography measurement into the model. It is found that the experimental and simulated values are similar under the conditions of natural fractures. Taking into account the hysteresis in the real situation, the experimental value is smaller than the simulated value. In general, the contact angle simulated by this model is feasible and the model is validated to undertake more parametric studies.

**Table 2:** Comparison of experimental and simulated contact angles.

Coal sample	Optical images	Simulated images
Hami lignite		
	45.9°	46.5°
Anyang coking coal		
	113.8°	114.1°
Zhaogu anthracite		
	82.2°	82.9°

## Parametric study and discussion

### Parametric study

**Figure 11:**
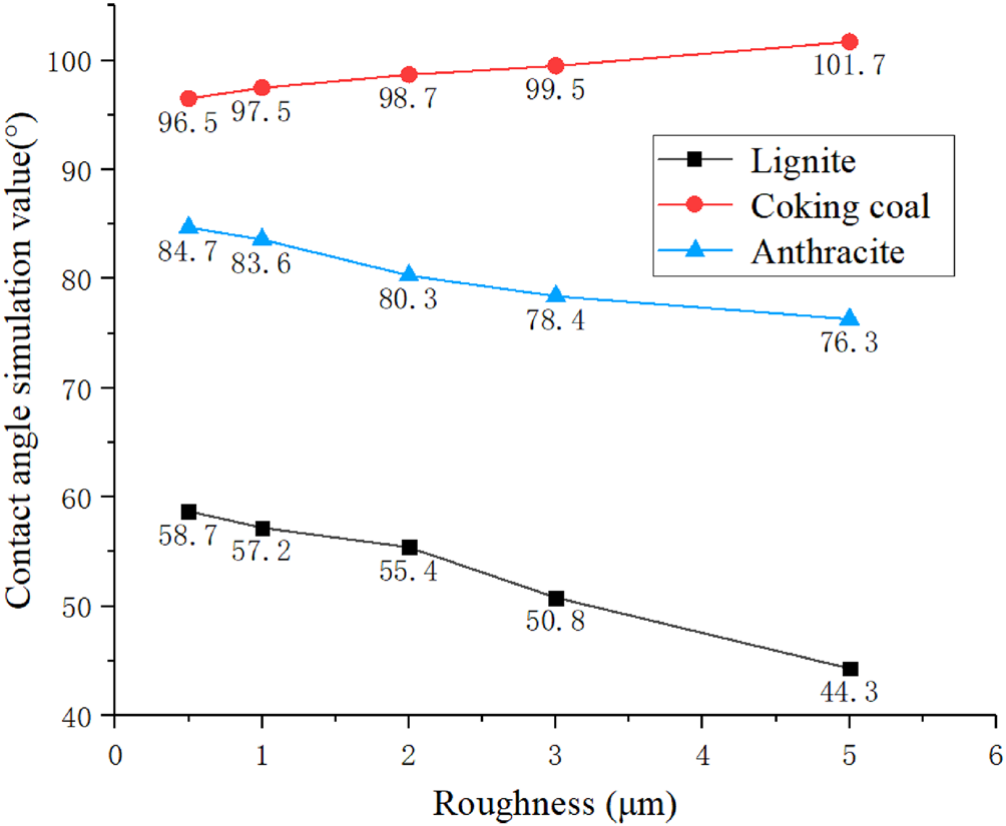
Simulated contact angles with different roughness of the three coal samples.

In the numerical simulation, the structural roughness of the surface is 0.5 μm, 1 μm, 2 μm, 3 μm and 5 μm, and the intrinsic contact angles of 60°, 85° and 95° are used as the research objects to simulate hydrophilic, weakly hydrophilic and hydrophobic of the wetting process. The contact angle of each coal sample surface is simulated. Figure 11 list the numerical results under three different wettability conditions and the trend of contact angle changes, respectively. From the simulation result image, it can be seen that as the roughness increases, the contact angle of the hydrophilic lignite surface gradually decreases, from 58.7° to 44.3°. As the roughness increases, its hydrophilicity becomes stronger. The change trend of hydrophobic coking coal is opposite to the previous two. As the roughness increases, the contact angle gradually increases, and its contact angle increases from 96.5° to 101.7°, becoming more hydrophobic. The change trend of the surface contact boundary of weakly hydrophilic anthracite is the same as that of lignite. Its contact angle reduces from 84.7° to 76.3°, and the hydrophilicity is improved. From the perspective of hydrophilicity and hydrophobicity, Hami lignite has the best hydrophilicity, and the influence of roughness on its wettability contact angle is about 14°. Anyang coking coal has the worst hydrophilicity, and the influence of roughness on the contact angle is about 6°. The Zhaogu anthracite is in the middle, with a variation range of about 8°.

### Discussion

**Figure 12:**
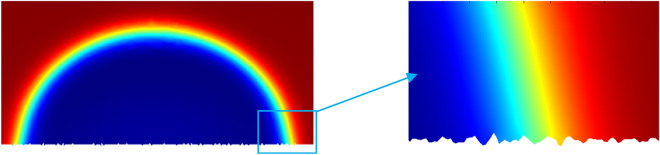
A close-up view of water-coal interface.

**Figure 13:**
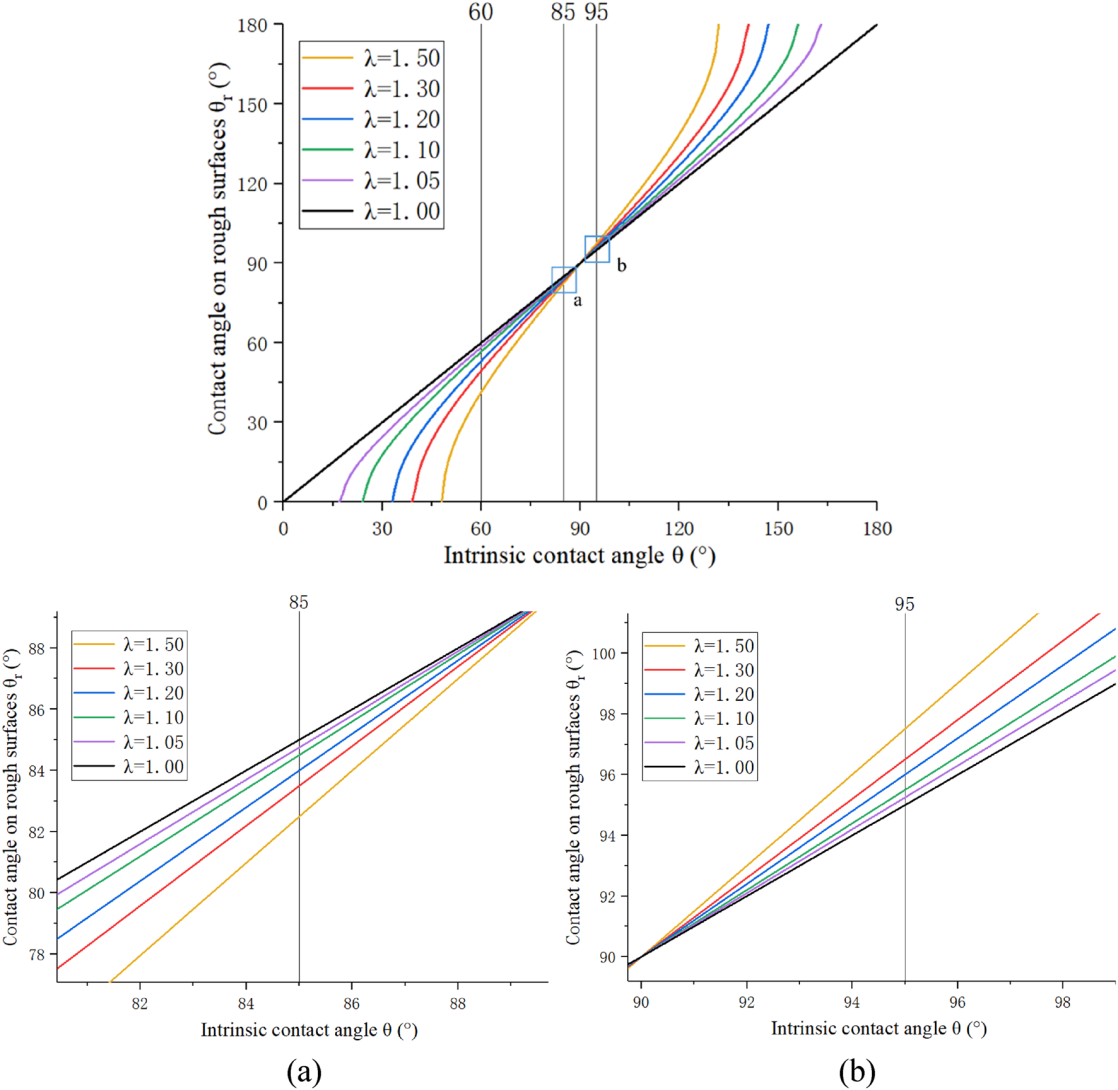
Wenzel model curve.

As shown in Fig. 12, the simulation result is that there is no air gap between the droplet and the solid, which conforms to the Wenzel model.

18 $$ cos{\theta_r} = \lambda \frac{{{\gamma_{sv}}{ - }{\gamma_{sl}}}}{{{\gamma_{lv}}}}{ = }\lambda cos\theta $$

where *λ* is always greater than 1, which is the ratio of the actual surface area to the projected area. In the roughness parameter design, the value of is 1.05, 1.1, 1.2, 1.3, 1.5. According to the Wenzel model, the theoretical curve of the relationship between the roughness factor and the contact angle is drawn. In Fig. 13, for the convenience of comparison, the curves with the intrinsic contact angles of 85° and 95° are partially zoomed in and listed as (a) and (b), respectively.

The Wenzel model can theoretically explain the above simulation results. For hydrophilic surfaces, when the contact angle is less than 90°, the contact angle gradually decreases as the surface roughness increases, and the hydrophilicity becomes stronger. For hydrophobic surfaces, when the contact angle is greater than 90°, the contact angle gradually increases as the roughness increases, and the hydrophobicity becomes stronger. A weakly hydrophilic surface has a smaller range of changes than a hydrophilic surface. For example, when the intrinsic contact angle is 60°, as the roughness factor increases, the contact angle decreases faster than the intrinsic contact angle of 85° decreases with the increase in roughness. This also explains, with the increase in roughness, that the change range of lignite contact angle is larger than that of anthracite.

**Figure 14:**
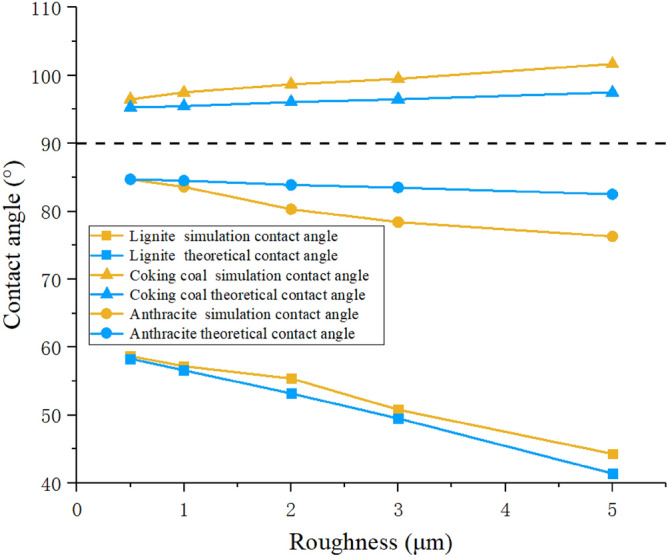
Comparison of simulated and theoretical contact angles.

The theoretical value of the apparent contact angles calculated according to the Wenzel theoretical model are compared with the simulated value, as shown in Fig. 14. Due to the local and global difference, the simulated value is the contact angle measured on the local area of the droplet, and the theoretical value is the contact angle calculated based on the entire rough surface area. When the contact angle is about 90°, the simulated value is compared with the theoretical value: the absolute value of the simulated contact angle is more than the theoretical value, and the contact angle change rate in the simulation is greater than the theoretical value. For hydrophilic surface, the simulated contact angle is less than the theoretical value. The simulation change law of the influence of the rough interface on the contact angle of the droplet accords with the Wenzel model with slight difference though.

## Conclusion

It is validated that the proposed numerical model is a feasible and efficient tool to investigate the effect of roughness on the wetting process of water on a solid matter surface. The model is then employed to study the wetting process of coal surfaces with different hydrophilic and hydrophobic properties under the influence of roughness, and the following conclusions are obtained:

1. The numerical simulation of roughness has a certain feasibility for the wetting of coal surface. The process of droplet spreading is similar to the real situation, including the speed of droplet spreading and droplet morphology. The contact angle values under the same roughness are similar. While the numerical simulation is too ideal, resulting in the simulated contact angle value being larger than the actual situation.
2. The wetting condition of the coal surface affected by the roughness conforms to the Wenzel model. The contact angle of hydrophilic lignite decreases with the increase of roughness, and the hydrophilicity is better. The contact angle of hydrophobic coking coal increases with the increase of roughness, and the hydrophobicity is better. The change in contact angle of weakly hydrophilic anthracite is between the two, and the difference in change is also in the middle.
3. Due to the local and total difference, the simulated value is the contact angle measured on the droplet range, and the theoretical value is the contact angle calculated based on the entire rough surface range. The simulated value of the contact angle is slightly different from the theoretical value obtained from the Wenzel model.

## Data Availability

All data generated or analysed during this study are included in this published article.
